# Bayesian spatio-temporal modeling of COVID-19 incidence in Algerian provinces using integrated nested Laplace approximations

**DOI:** 10.1038/s41598-025-28331-9

**Published:** 2025-12-04

**Authors:** Ayoub Asri

**Affiliations:** AIDAL Laboratory, Higher National School of Statistics and Applied Economics (ENSSEA), Kolea, Algeria

**Keywords:** Statistical model, COVID-19, Spatial analysis, Spatio-temporal analysis, Diseases, Health care, Mathematics and computing, Medical research

## Abstract

The COVID-19 pandemic in Algeria dissplayed significant spatial and temporal heterogeneity, especially during the severe summer 2021 wave driven by the Delta variant. Standard national-level statistics often obscure this critical local variation, creating a need for advanced modeling to inform precise public health interventions. This study aimed to perform a high-resolution spatio-temporal analysis of COVID-19 incidence across Algeria’s 48 provinces (Wilayas) to identify persistent high-risk areas and track the dynamics of viral spread. A spatio-temporal analysis was conducted on COVID-19 case data from all 48 Wilayas during epidemiological weeks 26-37 of 2021. We employed a Bayesian hierarchical model fitted using the Integrated Nested Laplace Approximation (INLA). The model incorporated structured spatial (Leroux prior), temporal (random walk of order 1), and spatio-temporal (Type IV interaction) random effects. Model selection was performed using the Watanabe-Akaike Information Criterion (WAIC) and Deviance Information Criterion (DIC) The spatio-temporally structured interaction model provided the best fit. Spatial heterogeneity was the dominant driver of transmission risk, accounting for 83.4% of the explained variance. Northeastern Wilayas, including Constantine and Tebessa, exhibited persistently high relative risks. The national temporal trend showed a sharp peak in early August 2021. The spatio-temporal interaction term (16.5% of variance) captured the progressive westward spread of the virus along the northern coast throughout the study period. This analysis demonstrates the critical utility of Bayesian spatio-temporal models in moving beyond national averages to identify specific high-risk areas and understand the evolving dynamics of an epidemic. The findings provide a valuable evidence base for designing targeted public health strategies. While this foundational study establishes the spatio-temporal risk patterns, future work incorporating socio-economic and environmental covariates will be essential to elucidate the underlying drivers of transmission.

## Introduction

The COVID-19 pandemic, caused by the SARS-CoV-2 virus, has presented an unprecedented global public health crisis, characterized by successive waves of infection driven by the emergence of new variants of concern^1^. In Algeria, the national experience mirrored this global trend, with distinct waves of infection and mortality observed over time. The available data, provided by the National Institute of Public Health (INSP), for instance, indicates the presence of multiple surges, including a prominent wave in mid-2021 and an even larger one in 2022^2^. These fluctuations highlight the dynamic and complex nature of disease spread, which is rarely uniform across a country. Understanding the spatial and temporal dimensions of these epidemic waves is crucial for informing public health policy and resource allocation^3^.

Standard aggregated national statistics, while useful for tracking overall trends, often obscure significant heterogeneity in disease spread at the local level^4^. Disease mapping, an essential statistical tool in epidemiology, addresses this limitation by estimating and visualizing disease risk across different geographical areas^5^. A major challenge in disease mapping is that raw estimates of the standardized mortality ratio (SMR), calculated as the ratio of observed to expected cases, can be highly unstable and unreliable, particularly for areas with small populations or a low number of events due to sampling variability^6^. Spatio-temporal analysis extends this framework by capturing how these risks evolve over time, allowing for the identification of “hotspots” of infection and providing a more nuanced understanding of disease dynamics^7^. This approach is particularly valuable for epidemics, where transmission is driven by a complex interplay of spatial and temporal factors^8^. By borrowing strength from neighboring areas and time points, these models can provide more reliable estimates of local disease risk, effectively smoothing the risk surface to produce more stable and reliable results^9^.

The objective of this study is to perform a detailed spatio-temporal analysis of new COVID-19 cases in Algeria’s 48 provinces (Wilayas) during the Summer 2021 wave. The analysis focuses on the period from late June to mid-September 2021, as defined by the available data (2021-06-25 to 2021-09-16). The study employs a Bayesian hierarchical modeling framework, implemented using the Integrated Nested Laplace Approximation (INLA) approach, to estimate Wilaya- and week-specific relative risks of infection^5^. The ultimate goal is to quantify the contributions of spatial, temporal, and spatio-temporal factors to the observed incidence patterns and to interpret these findings within the broader context of the public health situation in Algeria at that time.

### The Bayesian hierarchical framework for disease mapping

Disease mapping typically involves modeling count data, such as the number of new cases or deaths, using a Poisson distribution^10^. The number of observed cases in an area, $Y_i$, is modeled as a function of the expected number of cases, $E_i$, and a relative risk, $\theta _i$, such that the expectation is $E[Y_i]=E_i\theta _i$^10^.

To overcome the instability of raw SMRs, Bayesian hierarchical models treat the relative risks ($\theta _i$) as random variables and place prior distributions on them. By doing so, the models “borrow strength” from adjacent areas, effectively smoothing the risk estimates and producing more stable and reliable results^11^. The latent field in these models is often composed of a spatially structured component, which accounts for spatial autocorrelation due to shared risk factors or transmission pathways, and a spatially unstructured component, which captures unobserved local heterogeneity. The classical Besag-York-Mollié (BYM) model^12^ combines these two components but has been noted to suffer from a lack of identifiability and interpretability of its two variance parameters. While the BYM2 model^13^ offers a reparameterization that improves interpretability, we selected the alternative formulation proposed by Leroux et al. for this study.

The Leroux prior^14^ explicitly models a mixing parameter ($\lambda _s$) that governs the trade-off between spatially structured variation ($\lambda _s = 1$, reverting to the ICAR model) and unstructured, independent noise ($\lambda _s = 0$). This provides a direct and intuitive measure of the degree of spatial autocorrelation present in the data. The Leroux prior has been widely and successfully applied in spatio-temporal disease mapping for its flexibility and interpretability, including in studies of malaria^15^, hand-foot-mouth disease, and other infectious diseases^16^. While the stochastic partial differential equation (SPDE) approach^17^ is a powerful alternative for continuous spatial modeling, it was less suitable for thsis study, which utilizes aggregated areal data where the neighborhood structure is naturally defined by adjacency.

### The INLA approach as a modern tool for Bayesian inference

Traditionally, fitting complex Bayesian models required computationally intensive Markov Chain Monte Carlo (MCMC) methods, which could be slow and challenging to converge^5^. The Integrated Nested Laplace Approximation (INLA) has emerged as a powerful and computationally efficient alternative for a wide class of models known as latent Gaussian models (LGMs). INLA is a deterministic algorithm that provides accurate approximations to the posterior marginal distributions of the latent field and hyperparameters in a short computational time^18^. Its speed and flexibility make it an ideal tool for exploratory data analysis and for comparing a large number of competing model specifications, as is often required in complex spatio-temporal analyses^19^.

### Spatio-temporal modeling of COVID-19

Numerous studies have utilized spatio-temporal modeling to analyze the COVID-19 pandemic, consistently demonstrating that disease spread was highly heterogeneous across both space and time^20^. These analyses have shown that the transmission dynamics were driven by a complex interplay of socio-environmental, demographic, and public health factors^21^. For example, studies have identified positive associations between infection risk and variables such as population density, unemployment rate, and socioeconomic status. The heterogeneity of infection patterns underscores the need for localized public health interventions rather than national-level blanket policies. While the current study lacks these covariates, it serves as a foundational step by establishing the presence and magnitude of the spatio-temporal heterogeneity, thereby setting the stage for future research that can integrate such detailed determinants of risk. Other studies focused on Africa have also found that countries were affected to a heterogeneous extent and that disease spread was driven by complex spatial and temporal variations^22^.

A specific application in Algeria confirmed this spatial heterogeneity, identifying significant clusters of COVID-19 mortality in 2020, particularly in major urban centers like Algiers and Oran, using a Bayesian hierarchical model fitted with the Metropolis-Adjusted Langevin Algorithm (MALA)^23^.

### The evolution of the COVID-19 pandemic in Algeria: from initial outbreak to multiple waves

The COVID-19 pandemic in Algeria followed a pattern of multiple distinct waves, each with unique epidemiological characteristics and public health challenges. The virus was first confirmed to have reached Algeria in February 25, 2020^24^, initially spreading through imported cases from European countries such as Italy, France, and Spain^25^, with the first indigenous outbreak reported in the Wilaya of Blida on 1 March 2020, which became the initial epicenter of the epidemic^26^. The country experienced its first significant wave between March and June 2020, followed by subsequent waves of increasing intensity, particularly the devastating third wave in summer 2021.

#### Initial outbreak and early waves (2020)

Algeria’s first COVID-19 case was officially confirmed on February 25, 2020^25^. The early pandemic response included strict containment measures: the government implemented curfews, restricted gatherings, canceled public events, and issued stay-at-home orders between February and June 2020. The initial wave peaked in July 2020, with daily cases reaching approximately 700-800 infections. A second wave occurred in late 2020 and early 2021, though with less intensity than the devastating third wave that would follow^24^.

#### The summer 2021 third wave and delta variant impact

The summer 2021 wave in Algeria represented the country’s most severe epidemiological crisis of the pandemic. This period coincided with the global spread of the Delta variant (B.1.617.2), a highly transmissible strain that was first detected in India and accounted for approximately 71% of cases in Algeria during this period. The variant’s rapid transmission characteristics contradicted the previously held notion that viral spread would slow during warmer months^27^.

**Fig. 1:**
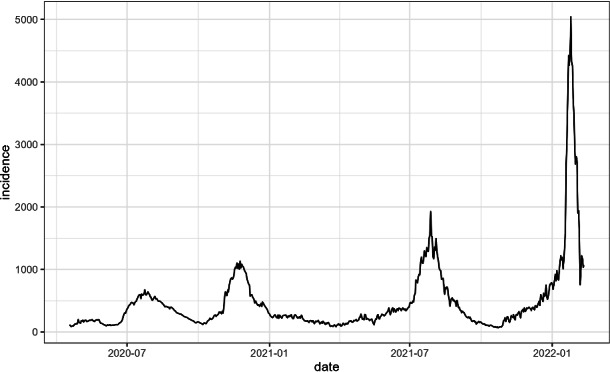
Historical timeline of COVID-19 incidence in Algeria.

As shown in Fig. 1, the summer 2021 wave demonstrated a dramatic surge in cases that far exceeded previous waves. The plot clearly shows four distinct waves, with the third wave (summer 2021) being particularly pronounced in both magnitude and duration. Epidemiological data indicates that daily cases skyrocketed from an average of 181–422 cases per day between January and June 2021 to a peak of 1927 cases on July 27, 2021—representing a five to tenfold increase^28^.

Similarly, daily deaths, which had remained below 10 through June 2021, reached tragic peaks of 49 and 46 deaths on July 28 and August 6, respectively^25^.

More details about the national situation are presented in Table 1^2,24,25^.

**Table 1:** Characteristics of COVID-19 waves in Algeria.

Wave period	Peak cases	Peak deaths	Dominant variant	Key characteristics
First wave (Mar–Jun 2020)	700–800/day	15–20/day	Original strain	Initial outbreak, strict containment measures
Second wave (late 2020-early 2021)	800–900/day	20–25/day	Alpha variant	Less intense than subsequent waves
Third wave (Jul–Aug 2021)	1,927/day	49/day	Delta variant (71 $\%$ of cases)$^{1}$	Most severe wave, rapid transmission
Fourth wave (late 2021)	1,200/day	30/day	Omicron variant	Vaccination moderating impact

National Institute of Public Health (INSP) statistics.

#### Public health response and vaccination challenges

Concurrent with the summer 2021 surge, the Algerian government was implementing a phased reopening plan in June and July 2021, which included the full return of public sector employees to work and the reduction of curfew hours^29^. In response to the dramatic surge in cases, containment measures were rapidly re-implemented, with a nighttime curfew extended to 14 Wilayas in July, and then to 37 Wilayas by early August^30^.

The national vaccination campaign, which began in January 2021, faced significant challenges during this period. By the end of August 2021, only 4.1 million doses had been administered in a population of approximately 45 million people^31^. Vaccine hesitancy was a major obstacle, with one study estimating the vaccine engagement rate at just 33.5% due to concerns about side effects (72.0%), demands for more efficacy and safety studies (48.3%), and beliefs in conspiracy theories (23.4%). By October 2021, only 27% of the target population had been fully vaccinated, and the overall percentage of the total population with at least one dose was just 17.88%^31^.

#### Spatiotemporal patterns and regional impact

The third wave exhibited distinct spatiotemporal patterns across Algeria’s 48 Wilayas. Northern and coastal Wilayas, being more densely populated and connected to international travel routes, were disproportionately affected during the initial phases of each wave. The spread typically followed a hierarchical diffusion pattern, beginning in major northern cities before spreading to smaller cities and eventually to the less populated southern regions. This pattern was particularly evident during the Delta-driven third wave, with early cases predominantly detected in Algiers and contiguous high-density Wilayas in the north of the country before spreading to other regions^32^.

The combination of a highly transmissible variant, a largely susceptible and unvaccinated population, and a phased return to normal social and economic activities created the conditions for a rapid and intense epidemic surge. The spatio-temporal model developed in this study provides a quantitative framework to analyze the statistical imprint of these events on the patterns of disease occurrence and identify high-risk regions for targeted interventions.

## Methods

### Study design and data preparation

Although Algeria’s new administrative division of 58 Wilayas was officially adopted on February 21st, 2021, the COVID-19 surveillance data for our study period (Summer 2021) was collected and reported using the previous 48-Wilaya structure. This temporal mismatch means data for the 10 newly created Wilayas were not available for 2021. To ensure consistency and avoid misalignment between geographic boundaries and historical case counts, our analysis was consequently conducted on these 48 spatial units. The adjacency matrix for the spatial random effect was constructed based on the boundaries of these 48 Wilayas as they existed during the study period

#### Study population and period

We conducted a national-level spatio-temporal epidemiological study utilizing surveillance data for COVID-19 from all 48 Algerian Wilayas. The analysis focused on a distinct wave of infection, spanning from June 25, 2021, to September 16, 2021 (epidemiological weeks 26 to 37). This period was selected to capture a self-contained epidemic phase with high incidence, minimizing the confounding effects of multiple, overlapping waves and varying public health interventions.

Furthermore, this period coincided with the emergence and dominance of the Delta variant in Algeria, allowing us to model the spatio-temporal dynamics of a specific viral lineage with uniform transmission characteristics. This aggregation also helps to smooth stochastic fluctuations and provides a more stable estimate of the underlying transmission rate.

#### Outcome variable and aggregation

The primary outcome was the daily count of new confirmed cases. To mitigate overdispersion, day-of-week reporting artifacts (e.g., lower weekend reporting), and to align with the latent timescale of epidemic spread, daily counts were aggregated into weekly incidence for each Wilaya.

#### Expected counts and indirect standardization

To account for heterogeneous population sizes and demographic structures across Wilayas, we calculated expected case counts. This process of indirect standardization estimates the number of cases expected in each Wilaya-week if its population experienced the national average risk. The expected counts ($E_{it}$) for area $i$ and week $t$ were calculated as $E_{it} = (\sum _t O_{it} / \sum _t P_i) * P_i$, where $O_{it}$ are the observed national cases in week $t$ and $P_i$ is the population of Wilaya $i$^33^.

The Standardized Mortality Ratio (SMR = Observed / Expected) provides a crude initial estimate of the relative risk but is unstable for small populations and does not borrow strength across space or time.

#### Geographic structure

**Fig. 2:**
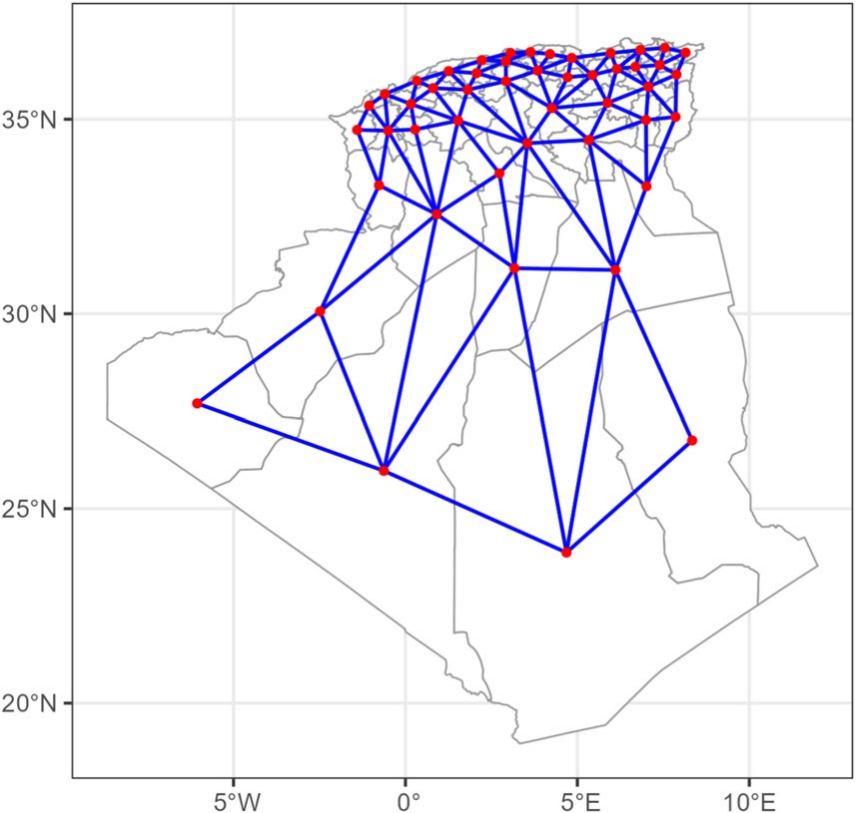
Spatial neighborhood structure of 48 Algerian Wilayas: first-order adjacency relationships.

A first-order neighbor adjacency graph was constructed for the 48 Wilayas. Two Wilayas were considered neighbors if they shared a common boundary. This graph shown in Fig. 2 defines the spatial dependency structure, formalized in a neighborhood matrix $W$ where diagonal elements are the number of neighbors and off-diagonal elements are -1 for adjacent areas. This matrix is the foundation of the conditional autoregressive (CAR) priors.

### Bayesian hierarchical modeling framework

We employed a Bayesian hierarchical model formulated as a generalized linear mixed model (GLMM) and implemented it using the Integrated Nested Laplace Approximation (INLA) algorithm. This approach is superior to maximum likelihood for complex random effects models as it provides a full posterior distribution for all parameters, naturally incorporating uncertainty.

The Bayesian paradigm is particularly suited for this task as it allows for the direct probabilistic interpretation of relative risks and exceedance probabilities, which are crucial for public health decision-making.

#### Likelihood

The observed weekly counts $O_{it}$ in area $i$ and week $t$ are assumed to follow a Poisson distribution with mean $\mu _{it}$:

$$\begin{aligned} O_{it} \sim \text {Poisson}(\mu _{it}),\;\;\;\;\;\mu _{it} = E_{it}.\theta _{it} \end{aligned}$$

where $\theta _{it}$ is the unknown relative risk. The model is formulated on the logarithmic scale:

$$\begin{aligned} \log (\theta _{it}) = \eta _{it} = \alpha + u_{i} + v_{t} + \gamma _{it} \end{aligned}$$

Here, $\eta _{it}$ is the linear predictor, and the components are:

$\alpha$ is the global intercept, representing the overall average log-relative risk. $u_i$ is the structured spatial random effect. $v_t$ is the structured temporal random effect. $\gamma _{it}$ is the spatio-temporal interaction random effect.

#### The INLA methodology: core principles

The Integrated Nested Laplace Approximation (INLA) is a deterministic method for approximate Bayesian inference, specifically designed for latent Gaussian models (LGMs). Unlike Markov Chain Monte Carlo (MCMC) methods, which rely on stochastic simulation, INLA uses nested Laplace approximations to compute accurate approximations of the marginal posterior distributions for the latent field and hyperparameters. This approach is computationally efficient and avoids convergence issues associated with MCMC, making it ideal for large spatio-temporal datasets^34^.

INLA is tailored for hierarchical models structured as follows:

1. Likelihood model: observations ${\bf y}=(y_1,...,y_n)$ are assumed conditionally independent given a latent Gaussian field ${\bf x}$ and hyperparameters $\boldsymbol{\theta }_1$, with density $\pi (y_i|x_i,\boldsymbol{\theta }_1)$.
2. Latent Gaussian field: the latent field ${\bf x}$ includes structured random effects (e.g., spatial, temporal, spatio-temporal) and fixed effects. It is assumed Gaussian with mean $\boldsymbol{\mu }$ and precision matrix ${\bf Q}(\boldsymbol{\theta }_2)$, i.e., ${\bf x}| \boldsymbol{\theta }_2 \sim \mathscr {N}(\boldsymbol{\mu }, {\bf Q}^{-1}(\boldsymbol{\theta }_2))$.
3. Hyperparameters: $\boldsymbol{\theta } = (\boldsymbol{\theta }_1,\boldsymbol{\theta }_2)$ govern the likelihood and latent field, with prior $\pi (\boldsymbol{\theta })$^35,36^.

The key posterior approximations in INLA involve:

- Marginal posterior for hyperparameters: $\pi (\theta _j|{\bf y}) \approx \int \pi (\boldsymbol{\theta }|{\bf y}) d \boldsymbol{\theta }_{-j}$ is approximated using a Laplace approximation $\tilde{\pi }(\boldsymbol{\theta }|{\bf y})$ evaluated over a grid $\{ \boldsymbol{\theta }_k \}$:
- Marginal posterior for latent field: $\pi (x_i|{\bf y}) \approx \int \pi (x_i|\boldsymbol{\theta },{\bf y})\pi (\boldsymbol{\theta }|{\bf y})d\boldsymbol{\theta }$ is computed by combining approximations conditional on $\boldsymbol{\theta }$:

$$\begin{aligned} \tilde{\pi }(x_i|{\bf y}) = \sum _k \tilde{\pi }(x_i|\boldsymbol{\theta }_k,{\bf y}) \tilde{\pi }(\boldsymbol{\theta }_k|{\bf y})\boldsymbol{\Delta }_k \end{aligned}$$

Here, $\tilde{\pi }(x_i|\boldsymbol{\theta }_k,{\bf y})$ is obtained via Laplace approximation or simplified Laplace approximation for improved accuracy^18^.

For our model, the latent field ${\bf x}=\{\boldsymbol{\alpha },\boldsymbol{u},\boldsymbol{v},\boldsymbol{\gamma }\}$ includes all random effects, and hyperparameters $\boldsymbol{\theta }$ include precision parameters $\tau _u,\tau _v,\tau _\gamma$ and spatial smoothing parameter $\lambda _s$. INLA efficiently computes approximations for $\pi (\theta _{it}|\boldsymbol{O})$ and $\pi (\tau _u|\boldsymbol{O})$, etc., without MCMC sampling.

#### Why INLA? Advantages over MCMC for this study

INLA was chosen over MCMC for several compelling reasons:

- Computational efficiency: INLA reduces computation time from hours (typical for MCMC) to minutes for large spatio-temporal models. This efficiency was crucial given our dataset of 48 Wilayas $\times$ 12 weeks = 576 observations and complex random effects.
- Accuracy: for LGMs, INLA provides approximations with high accuracy, often comparable to MCMC. Our use of the simplified Laplace approximation ensured precision in posterior marginals.
- Avoidance of convergence issues: MCMC requires careful diagnostics (e.g., trace plots, Gelman-Rubin statistics) to ensure convergence. INLA, being deterministic, eliminates these concerns.
- Implementation flexibility: the R-INLA package supports a wide range of latent models (e.g., CAR, RW1, RW2) and interaction types. This allowed us to rigorously test multiple temporal and spatio-temporal structures.

INLA is particularly suited for epidemiology, as demonstrated in studies on dengue^37^ and COVID-19^38^, where it efficiently handled spatial and temporal autocorrelation.

#### Random effects specification and priors (the heart of the model)

Spatial component ($u_i$): Leroux prior

We chose the Leroux prior, a generalization of the intrinsic CAR (ICAR) and independent random effects models. Its conditional distribution is:

$$\begin{aligned} u_i|{\bf u}_{-i},\lambda _s,\tau _u \sim \mathscr {N} \left( \frac{\lambda _s \sum _{j \sim i}u_j}{\lambda _s n_i+1-\lambda _s}, \frac{1}{\tau _u(\lambda _s n_i+1-\lambda _s)} \right) \end{aligned}$$

where $n_i$ is the number of neighbors of area $i$. The hyperparameter $\lambda _s \in [0,1]$ controls the blend between spatial smoothing ($\lambda _s = 1$, reverting to ICAR) and independent noise ($\lambda _s = 0$). This flexibility allows the data to dictate the degree of spatial smoothing required.

Vague (non-informative) priors were assigned:

- Precision $\tau _u$: $p(\tau _u) \propto 1/\tau _u$ (a uniform prior on the standard deviation $\sigma _u$).
- Smoothing $\lambda _s$: $\lambda _s \sim \text {Beta}(1, 1)$ (Uniform(0,1)). To assess the sensitivity of our results to prior choice, we also considered a penalized complexity (PC) prior for the spatial standard deviation $\tau _u$, which penalizes deviation from a baseline model of smoothness^39^. The results were robust to this alternative specification.

Temporal component ($v_t$): random walk priors

We evaluated two priors for the temporal trend:

- RW1 (first order random walk): assumes a constant evolution between consecutive time points, $v_t | v_{t-1} \sim N(v_{t-1}, \tau _v^{-1})$. Its precision matrix $Q_{\gamma _{RW1}}$ is derived from first-order differences.
- RW2: assumes smoothness by enforcing consistency in the second-order differences, $v_t | v_{t-1}, v_{t-2} \sim N(2v_{t-1} - v_{t-2}, \tau _v^{-1})$. This yields a smoother trend, less sensitive to short-term fluctuations.

PC priors were also considered for the temporal precision parameters.

Spatio-temporal interaction ($\gamma _{it}$): Knorr–Held typology

We rigorously tested all four interaction types proposed by Knorr-Held et al.^40^, which represent different philosophical assumptions about the dependency structure:^41^

- Type I (unstructured interaction): $\gamma _{it} \sim N(0, \tau _\gamma ^{-1})$. Assumes no structure; interactions are independent noise. The least complex model.
- Type II (temporally structured interaction): $\boldsymbol{\gamma }_t \sim \text {CAR}({\bf Q}_{xi}, \tau _\gamma )$ and $\boldsymbol{\gamma }_t$ evolves over time according to a temporal process (in our case RW1 or RW2). This implies the spatial pattern evolves smoothly over time.
- Type III (spatially structured interaction): $\boldsymbol{\gamma }_i \sim \text {RW1}(\tau_\gamma )$ and this temporal pattern varies smoothly across the spatial neighborhood structure. This implies the temporal trend is similar in adjacent areas.
- Type IV (fully structured interaction): the interaction is structured in both space and time. The precision matrix is given by the Kronecker product of the temporal and spatial precision matrices, ${\bf R} = {\bf Q}_{\text {time}} \otimes {\bf Q}_{xi}$. This is the most parsimonious and complex model, assuming a spacetime process where dependence decays with both spatial and temporal distance.

#### Identifiability and constraints

Gaussian Markov random fields (GMRFs) like RW and CAR are inherently improper (intrinsic). To ensure identifiability, a sum-to-zero constraint was applied to each set of random effects: $\sum _i u_i = 0$, $\sum _t v_t = 0$, $\sum _i \gamma _{it} = 0 \forall t$, $\sum _t \gamma _{it} = 0 \forall i$. These constraints are automatically handled in the implementation.

### Model implementation, selection, and inference

It is important to note that the current model focuses on estimating the underlying spatio-temporal risk structure without including specific covariates (e.g., population density, mobility, socioeconomic factors). This serves as a crucial foundational step to first identify and map the robust patterns of risk, which in turn generates hypotheses about potential drivers that can be tested in future studies with integrated covariate data.

#### Implementation in INLA

Models were fitted using the R-INLA package^42^. The INLA algorithm operates in three key steps:

1. Grid construction: a grid of values $\{ \boldsymbol{\theta }_k \}$ is constructed for the hyperparameters.
2. Laplace approximation: for each $\boldsymbol{\theta }_k$, approximate the conditional posterior $\pi ({\bf x}|\boldsymbol{\theta }_k,{\bf y})$ using a Gaussian Laplace approximation.
3. Numerical integration: marginal posteriors are obtained by integrating over $\boldsymbol{\theta }$ using numerical methods.

Convergence and accuracy of these approximations were assessed by examining the stability of the posterior distributions across successive iterations of the approximation process. Furthermore, the internal diagnostics inherent to the INLA algorithm were consulted to ensure that the approximations were robust and that the numerical integration over the hyperparameter space was sufficiently dense to provide stable and reliable results. This comprehensive approach ensures that the inferred posterior distributions are a faithful representation of the true, but intractable, Bayesian posteriors.

#### Model selection

We employed a comprehensive suite of Bayesian model selection criteria to compare the eight candidate models (4 interaction types and 2 temporal priors):

- Deviance information criterion (DIC): balances model fit and complexity, but can be unstable for complex hierarchical models.
- Watanabe-akaike information criterion (WAIC): a more fully Bayesian measure of out-of-sample predictive accuracy.
- Logarithmic score (LS): calculated from the conditional predictive ordinate (CPO). It directly measures the model’s predictive quality, with lower scores indicating better performance.

The model with the lowest WAIC and LS was selected as the final model for inference. We also inspected the posterior mean of the log-CPO (and the probability integral transform, PIT) to check for overall model calibration and to identify any potential outliers or areas of poor fit.

#### Posterior inference

After selecting the best-fitting model, we extracted the following from the posterior distributions:

- Posterior medians: of the relative risks $\theta _{it}$ for mapping.
- 95% credible intervals (CrI): for the relative risks, quantifying uncertainty.
- Exceedance probabilities: $P(\theta _{it} > 1 | \text {data})$. A probability $>0.8 \;\mathrm{or} >0.9$ is often used to flag areas with significantly elevated risk.
- Variance decomposition: the proportion of total variance in the linear predictor $\eta _{it}$ attributable to the spatial ($u_i$), temporal ($v_t$), and interaction ($\gamma _{it}$) components was calculated to quantify their relative importance.

Additionally, we extracted the posterior distributions of the hyperparameters (e.g., $\lambda _s$, $\tau _u$) to interpret the degree of spatial smoothing and the magnitude of the random effects.

## Results

This study employed Integrated Nested Laplace Approximation (INLA) within a Bayesian framework to model the spatio-temporal dynamics of COVID-19 incidence across Algeria’s 48 Wilayas during the third wave of the pandemic, which was identified as occurring between June 25th and September 16th, 2021 (epidemiological weeks 26 to 37).

A full comparison of all eight candidate models based on the Deviance Information Criterion (DIC), effective number of parameters, Mean CPO, Logarithmic score and the Watanabe-Akaike Information Criterion (WAIC) is provided in Table 2. Based on the DIC and WAIC criteria (where lower values indicate better models), TypeIV.RW1 appears to be the best performing model with the lowest WAIC and a competitive DIC. The other models, particularly TypeII.RW2 and TypeIV.RW2, show significantly poorer performance across multiple metrics, with TypeII.RW2 having the highest values in DIC (4501.241), WAIC (4992.547), and LS (6774.666), making it the least favorable option.

**Table 2:** Comparative performance of eight competing spatio-temporal models for COVID-19 incidence in 48 Algerian Wilayas during summer 2021: DIC, WAIC, LS, and CPO metrics.

Model	Mean deviance	$\hbox {effective}^{1}$	DIC	WAIC	$\hbox {LS}^{2}$	Mean CPO
TypeI.RW1	3722.670	500.5264	4223.196	4158.594	5033.491	0.0177
TypeII.RW1	3783.898	453.6289	4237.527	4232.695	3686.943	0.0136
TypeIII.RW1	3762.147	493.6173	4255.764	4217.465	4932.483	0.0106
TypeIV.RW1	3722.494	500.5419	4223.036	4158.306	5030.571	0.0176
TypeI.RW2	3758.839	455.5798	4214.419	4188.046	3534.017	0.0133
TypeII.RW2	3937.562	563.6782	4501.241	4992.547	6774.666	0.0058
TypeIII.RW2	3762.150	493.6177	4255.768	4217.475	4932.565	0.0106
TypeIV.RW2	3848.858	451.7473	4300.605	4383.269	4734.032	0.0082

Effective number of parameters. logarithmic score.

### Model selection

To capture the underlying risk structure, we compared eight different models resulting from the combination of four interaction types (I–IV) and two prior specifications for the temporal random effect (first-order and second-order random walk, RW1 and RW2). Model performance was evaluated using the Deviance Information Criterion (DIC) and the Watanabe-Akaike information criterion (WAIC).

The model with the best fit to the data was the Type IV interaction model with a RW1 prior for the temporal effect (DIC = 4237.52, WAIC = 4232.68). This model incorporates a spatially structured area effect, a temporally structured random effect, and an interaction term that is itself spatially and temporally structured. This complex structure suggests that the excess risk during this period was influenced by intrinsic spatial patterns, a strong temporal trend, and an interaction that evolved in both space and time.

### Parameter estimates

The posterior mean and standard deviation for the key parameters of the selected Type IV RW1 model are presented in Table 3.

**Table 3:** Posterior parameter estimates for the selected Bayesian spatio-temporal model of COVID-19 incidence across 48 Algerian Wilayas, summer 2021.

Parameter	Description	Mean	SD
$\alpha$	Intercept	-0.792	0.023
$\sigma ^2_s$[1]	Spatial variance	3.927	1.514
$\lambda _s$	Spatial smoothing	0.423	0.193
$\sigma ^2_t$[2]	Temporal variance	0.005	0.004
$\sigma ^2_{st}$[3]	Spatio-temporal variance	1.105	0.101

Inverse of precision : $1/\tau _u$, inverse of precision : $1/\tau _v$, inverse of precision : $1/\tau _{\gamma }$.

The high value of the spatial variance ($\sigma ^2_s$) compared to the temporal variance ($\sigma ^2_t$) indicates substantial heterogeneity in COVID-19 risk between Wilayas that is consistent over time. The spatio-temporal interaction variance ($\sigma ^2_{st}$) is also considerable, confirming that the spatial pattern of risk was not static but changed throughout the wave.

### Decomposition of variability

The relative contribution of the structured spatial, temporal, and spatio-temporal components to the overall variability in the relative risks was calculated. The spatial component accounted for 83.4% of the explained variability, the spatio-temporal interaction for 16.5%, and the pure temporal component for only 0.2%. This decomposition underscores that geographical factors were the dominant drivers of the observed incidence patterns during the third wave, while the overall temporal trend, though present, was relatively uniform across the country.

### Spatial pattern of risk

**Fig. 3:**
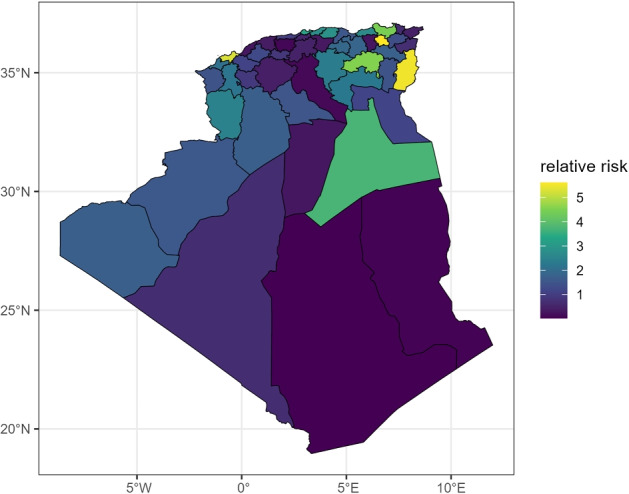
Spatial distribution of COVID-19 relative risk across 48 Algerian Wilayas, summer 2021: posterior median estimates from Bayesian model.

The posterior median of the spatially structured relative risk (RR) for each Wilaya, $\exp {(u_i)}$, is displayed in Fig. 3. This map represents the stable, area-specific component of risk, adjusted for the national temporal trend.

Wilayas in the northern-central and eastern regions, such as Constantine, Sétif, and Tebessa, exhibited persistently high endemic relative risks ($\hbox {RR}>1.5$). Conversely, the vast southern Wilayas (e.g., Adrar, Tamanghasset, Tindouf) and some coastal areas showed lower-than-average stable risks ($\hbox {RR}<1$).

**Fig. 4:**
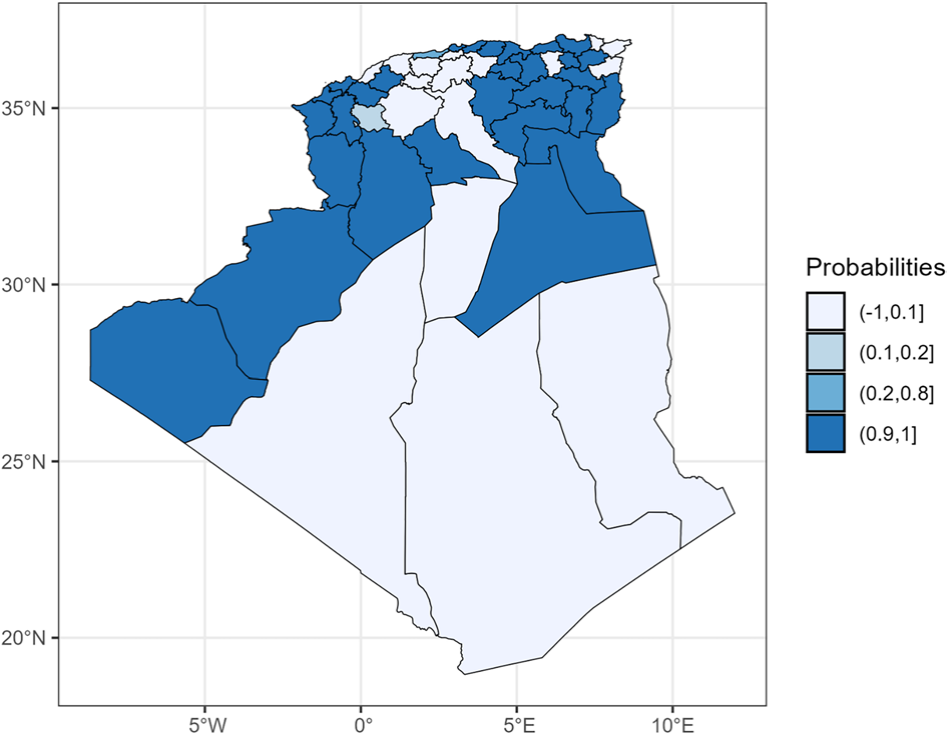
Statistical significance of COVID-19 risk in Algerian Wilayas: posterior relative risk exceedance probability, summer 2021.

Figure 4 shows the posterior probability that the spatial relative risk exceeded 1. Wilayas with probabilities greater than 0.8 (dark blue) can be considered to have a significantly elevated endemic risk with high certainty.

### Temporal pattern of risk

**Fig. 5:**
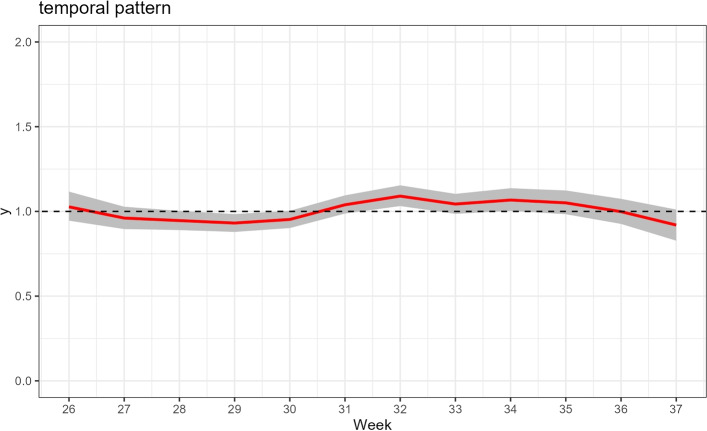
National temporal trend of COVID-19 relative risk in Algeria during summer 2021 delta variant wave (weeks 26-37).

The national temporal trend of the relative risk, $\exp (v_t)$, is shown in Fig. 5. The trend shows a clear progression of the wave: a sharp increase in risk starting around week 29 (mid-July), peaking around week 32–33 (early August), followed by a steady decline until the end of the study period in week 37 (mid-September). The credibility interval narrows at the peak, indicating higher certainty in the model’s estimate during the height of the wave.

### Spatio-temporal evolution of the epidemic

**Fig. 6:**
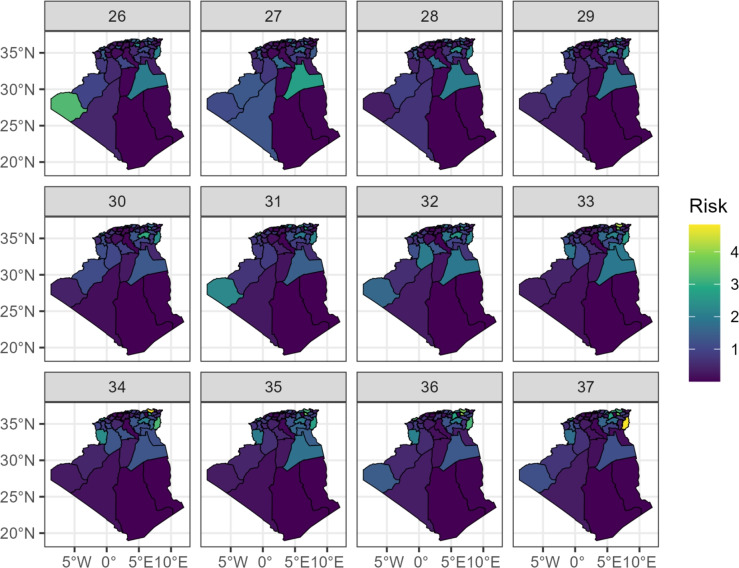
Spatio-temporal evolution of COVID-19 relative risk across 48 Algerian Wilayas: weekly posterior median estimates, summer 2021.

**Fig. 7:**
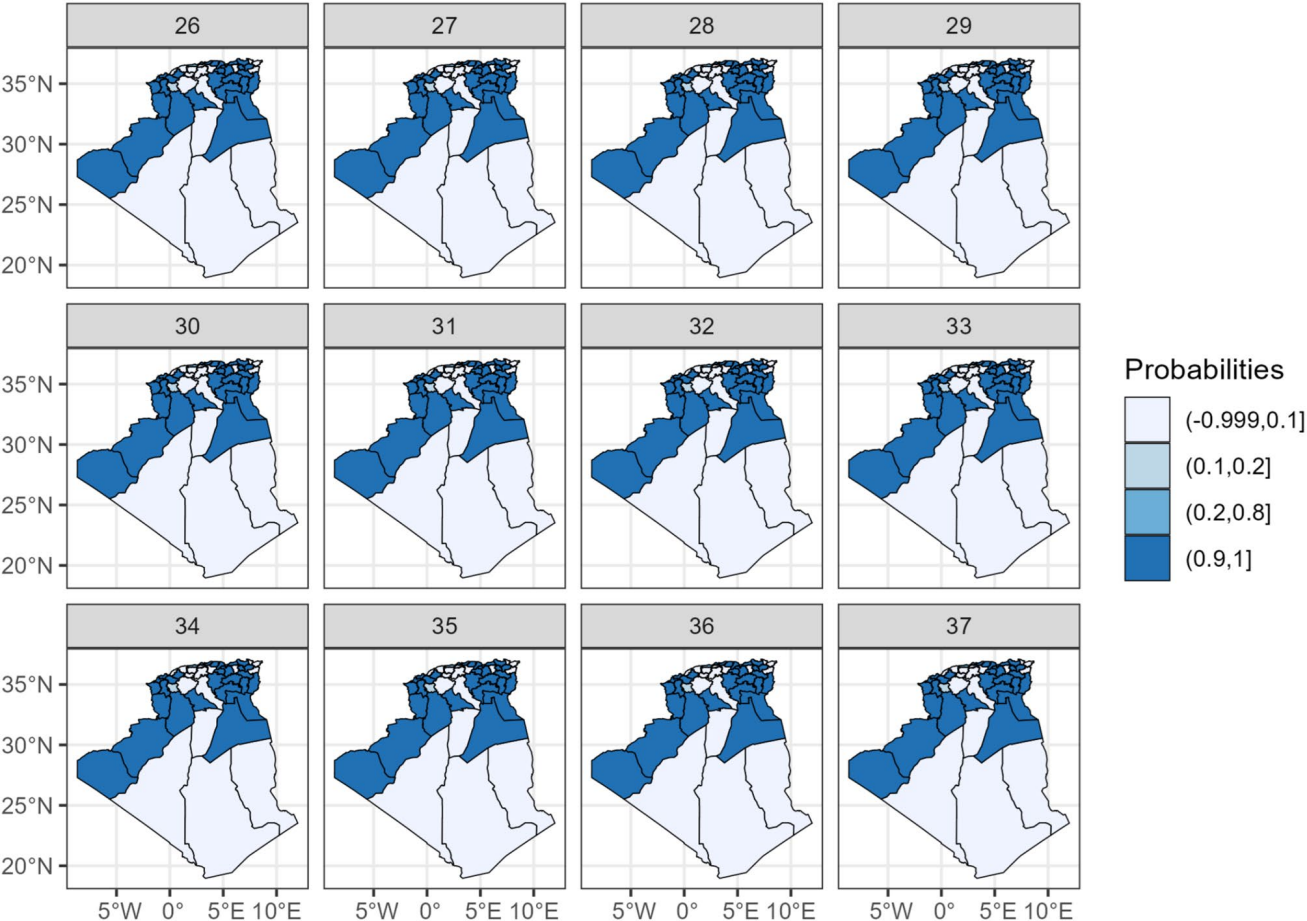
Weekly progression of statistically significant COVID-19 risk across Algerian Wilayas: posterior exceedance probability maps, summer 2021.

The evolution of the relative risk for each Wilaya throughout the 12-week period is visualized in Fig. 6 and 7. Figure 6 maps the posterior median RR for each week, while Fig. 7 maps the posterior probability that the RR exceeds 1.

These maps reveal the dynamic spread of the virus: Initial Phase (Weeks 26–28): High risks are already apparent in the northeastern Wilayas (e.g., Skikda, Guelma, El Tarf) and a few scattered central ones. Growth and Peak Phase (Weeks 29–34): The high-risk area expands dramatically westward and southward along the northern belt. By week 32, most northern Wilayas from Annaba in the east to Tlemcen in the west are experiencing peak risks. The central Wilayas of Djelfa and Msila also show a significant increase. Decline Phase (Weeks 35–37): While the overall risk decreases nationally, the spatial heterogeneity remains. The eastern regions (e.g., Tebessa, Khenchela) and some central Wilayas maintain elevated risks longer than the western regions.

### Area-specific trajectories

The model’s estimated relative risks (RR) and the raw standardized morbidity ratios (SMR = Observed/Expected) for a selection of Wilayas are plotted in the next Figures. These plots highlight the model’s ability to smooth unstable SMR estimates based on sparse data (borrowing strength from neighboring areas and time points) and provide credible intervals.

**Fig. 8:**
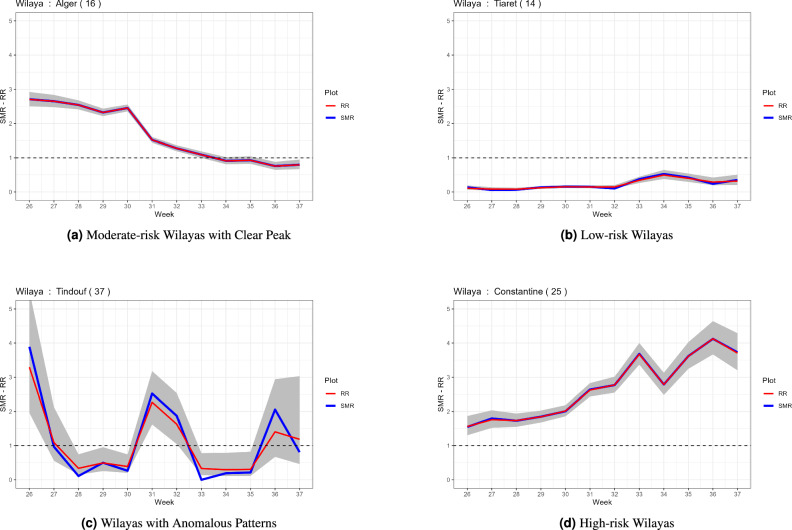
Area-specific temporal trends of COVID-19 incidence during the Summer 2021 wave in Algeria. (**a**) Moderate-risk Wilayas, often major urban centers, showed a clear peak-and-decline pattern. (**b**) Low-risk Wilayas, primarily in the south, had relative risks at or below 1. (**c**) Wilayas with anomalous patterns where model estimates diverged from raw SMRs. (**d**) High-risk Wilayas exhibited consistently elevated relative risks throughout the study period.

- High-risk Wilayas (Fig. 8d): This category is defined by a consistently elevated risk throughout the study period. The Relative Risk (RR) and Standardized Morbidity Ratio (SMR) values remain significantly above 1, indicating a sustained and high incidence of cases. The model’s smoothed RR trajectory closely follows the raw SMR, confirming the persistent and severe outbreak in these areas.

The Wilayas in this category are Tebessa, Constantine, Skikda, Batna, Oran, Ouargla, Tizi Ouzou and Aïn Témouchent.

- Moderate-risk Wilayas with clear peak (Fig. 8a): characteristic of major population centers, these Wilayas exhibit a strong, bell-shaped temporal pattern. The epidemic wave is marked by a rapid rise to a sharp peak, followed by a steady decline. The model effectively captures this distinct peak-and-decline pattern, demonstrating its ability to track intense, time-limited outbreaks.

This pattern was observed in the major urban centers of Wilayas Alger, Tipaza, Boumerdès, Béjaia, Bordj Bou Arréridj, Sétif, M’Sila, Tlemcen, Saïda, Béchar, El Oued, Guelma, Khenchela, Laghouat, Oum el Bouaghi, Sidi Bel Abbès, Adrar and Relizane.

- Low-risk Wilayas (Fig. 8b): These areas experienced a comparatively mild epidemic wave, with RR and SMR estimates consistently centered at or below 1. The model’s credibility intervals are often wider here, reflecting the greater uncertainty in estimates due to lower population densities and fewer reported cases.

This group primarily includes southern Wilayas such as Tamanghasset, Adrar ,Aïn Défla, Annaba, Biskra, blida, Bouira, Chlef, Djelfa, Mascara, Mila, Mostaganem, Médéa, Souk Ahras, El Tarf, Ghardaïa, Tiaret and Illizi.

- Wilayas with anomalous patterns (Fig. 8c): This category captures areas where the model’s smoothed estimate diverges notably from the raw data. A high SMR coupled with a lower, smoothed RR suggests a highly localized or outlier event. The model’s spatial smoothing structure, which borrows information from neighboring areas, attenuates these extreme local estimates towards the regional mean.

Wilayas such as El Bayadh, Jijel, Naâma, Tissemsilt, and Tindouf displayed this anomalous pattern.

In summary, the spatio-temporal analysis revealed that the third COVID-19 wave in Algeria was primarily driven by strong geographical factors, with a clear national temporal trajectory. The epidemic originated and was most intense in the northeastern regions before spreading westwards along the populated northern coast, with the central and southern regions remaining significantly less affected.

## Discussion

This study provides a comprehensive analysis of the spatio-temporal dynamics of COVID-19 incidence in Algeria during the devastating third wave of Summer 2021. By employing a Bayesian hierarchical model with INLA, we moved beyond unstable raw SMRs to obtain smoothed, reliable estimates of relative risk that account for both spatial and temporal dependencies.

Our key finding is the overwhelming dominance of spatial structure (83.4% of explained variance) in driving transmission risk during this period. The identified high-risk corridor in the northern-central and eastern Wilayas (e.g., Constantine, Setif, Tebessa) aligns with known demographic and infrastructural factors. These regions are characterized by higher population densities, greater connectivity through major transportation networks, and increased economic activity, all of which facilitate virus transmission. The significantly lower risk in the vast southern Wilayas underscores the role of low population density and relative isolation as protective factors.

The chosen best-fitting model (Type IV interaction with RW1 temporal effect) indicates that the epidemic evolution was not merely a function of a national trend and static spatial risks. Instead, the significant spatio-temporal interaction (16.5% of variance) captures how the spatial pattern of risk itself evolved over time. Our results visually demonstrate a classic hierarchical diffusion pattern, beginning in the northeast before spreading westward along the populated northern coast. This pattern is consistent with the initial importation of cases through international travel to northern hubs, followed by domestic spread via road networks and social contacts, as noted in other studies of COVID-19 diffusion.

The temporal trend itself, while clear and statistically significant, accounted for a minimal portion of the variance (0.2%). This suggests that while the entire country experienced the same overarching wave pattern—a rapid rise, peak, and decline primarily driven by the transmissibility of the Delta variant and the timing of public health measures—the actual experience of risk for a citizen was far more dependent on their location than on the national week-to-week progression.

## Conclusions

This study successfully applied a sophisticated Bayesian spatio-temporal model to dissect the complex dynamics of the COVID-19 pandemic in Algeria during the intense Summer 2021 wave. The analysis provides several key takeaways:

1. Dominance of spatial heterogeneity: the primary driver of transmission risk was spatial, accounting for over 83% of the explained variance. This underscores that an individual’s risk was determined more by their geographical location than by the national progression of the wave. The identification of a persistent high-risk corridor in the northern-central and eastern Wilayas (e.g., Constantine, Sétif, Tebessa) highlights areas of enduring vulnerability, likely due to factors like population density, connectivity, and socioeconomic activity.
2. Clear diffusion pattern: the spatio-temporal interaction, which accounted for a significant portion of the remaining variance, revealed a distinct hierarchical diffusion pattern. The wave originated in the northeastern regions before systematically propagating westward along the populated northern coast. This pattern is consistent with the importation of the Delta variant through international nodes followed by domestic spread via major transportation networks and social contacts.
3. Validation of modeling approach: the Bayesian hierarchical framework implemented via INLA proved highly effective. It provided stable, smoothed estimates of relative risk by borrowing strength from neighboring areas and time points, overcoming the instability of raw SMRs, especially in low-population Wilayas. The selection of a Type IV interaction model confirms that the underlying process was structured in both space and time, requiring a complex model for accurate representation.
4. Public health implications: these findings strongly advocate for a localized, pre-emptive approach to public health intervention. Rather than uniform national policies, resources and measures (e.g., testing, vaccination campaigns, healthcare readiness) should be prioritized for the identified high-risk spatial hubs, particularly at the beginning of a wave. Understanding the typical diffusion pathway allows authorities in western and southern regions to anticipate the arrival of a wave from the northeast and enact timely containment measures.

In summary, this analysis moves beyond national-level statistics to reveal the profound and structured geographical inequality in COVID-19 impact across Algeria. It provides a robust methodological framework and a detailed empirical map of risk, serving as a valuable foundation for targeted public health strategy and future research into the specific socio-environmental determinants driving these spatial patterns.

## Data Availability

The current data and code are publicly available at: https://github.com/ayoub-asri/covid_article

## References

[1] El-Shabasy, R. M. et al. Three waves changes, new variant strains, and vaccination effect against COVID-19 pandemic. *Int. J. Biol. Macromol.* **204**, 161–168 (2022). 35074332 10.1016/j.ijbiomac.2022.01.118 PMC8782737

[2] INSP. INSP COVID-19 epidemiological situation. https://www.insp.dz/index.php/publications/situation-epidemiologique-covid19.html (2021). Accessed 30 Dec 2024.

[3] Huang, J. & Morris, J. S. Infectious disease modeling. *Annu. Rev. Stat. Appl.* **12**, 19–44 (2025).

[4] Zhang, Y., Britton, T. & Zhou, X. Monitoring real-time transmission heterogeneity from incidence data. *PLoS Comput. Biol.* **18**, e1010078 (2022). 36455043 10.1371/journal.pcbi.1010078 PMC9746975

[5] Schrödle, B. & Held, L. Spatio-temporal disease mapping using INLA. *Environmetrics* **22**, 725–734 (2011).

[6] Rezaeian, M., Dunn, G., St Leger, S. & Appleby, L. Geographical epidemiology, spatial analysis and geographical information systems: A multidisciplinary glossary. *J. Epidemiol. Commun. Health* **61**, 98–102 (2007). 10.1136/jech.2005.043117 PMC2465628 17234866

[7] Guerrero, B. V. et al. Assessing the spatio-temporal risk of aedes-borne arboviral diseases in non-endemic regions: The case of northern Spain. *PLoS Negl. Trop. Dis.* **19**, e0013325 (2025). 40720537 10.1371/journal.pntd.0013325 PMC12313078

[8] Rodriguez-Idiazabal, L. et al. Understanding the COVID-19 pandemic through Bayesian spatio-temporal modeling of several outcomes. *Spat. Spatiotemp. Epidemiol.* **54**, 100737 (2025). 10.1016/j.sste.2025.100737 40935505

[9] Waller, L. A. & Carlin, B. P. Disease mapping. *Chapman Hall CRC Handb. Mod. Stat. Methods* **2010**, 217–243 (2010). 10.1201/9781420072884-c14 PMC4180601 25285319

[10] Mohebbi, M., Wolfe, R. & Forbes, A. Disease mapping and regression with count data in the presence of overdispersion and spatial autocorrelation: A Bayesian model averaging approach. *Int. J. Environ. Res. Public Health* **11**, 883–902 (2014). 24413702 10.3390/ijerph110100883 PMC3924480

[11] Richardson, S., Thomson, A., Best, N. & Elliott, P. Interpreting posterior relative risk estimates in disease-mapping studies. *Environ. Health Perspect.* **112**, 1016–1025 (2004). 15198922 10.1289/ehp.6740 PMC1247195

[12] Besag, J., York, J. & Molli, A. Bayesian image restoration, with two applications in spatial statistics. *Ann. Inst. Stat. Math.* **43**, 1–20 (1991).

[13] Riebler, A., Sørbye, S. H., Simpson, D. & Rue, H. An intuitive Bayesian spatial model for disease mapping that accounts for scaling. *Stat. Methods Med. Res.* **25**, 1145–1165 (2016). 27566770 10.1177/0962280216660421

[14] Leroux, B. G., Lei, X. & Breslow, N. *Estimation of disease rates in small areas: A new mixed model for spatial dependence*. 179–191. In The IMA Volumes in Mathematics and Its Applications (Springer, 2000).

[15] Kitawa, Y. S., Johnson, O., Giorgi, E. & Asfaw, Z. G. Understanding the importance of spatial correlation in identifying spatio-temporal variation of disease risk, in the case of malaria risk mapping in Southern Ethiopia. *Sci. Afr.* **22**, e01926 (2023).

[16] Du, Z. et al. Bayesian spatiotemporal analysis for association of environmental factors with hand, foot, and mouth disease in Guangdong, China. *Sci. Rep.* **8**, 15147 (2018). 30310172 10.1038/s41598-018-33109-3 PMC6181968

[17] Lindgren, F., Rue, H. & Lindström, J. An explicit link between gaussian fields and gaussian Markov random fields: The stochastic partial differential equation approach. *J. R. Stat. Soc. Ser. B Stat. Methodol.* **73**, 423–498 (2011).

[18] Rue, H., Martino, S. & Chopin, N. Approximate Bayesian inference for latent gaussian models by using integrated nested Laplace approximations. *J. R. Stat. Soc. Ser. B Stat. Methodol.* **71**, 319–392 (2009).

[19] Bivand, R. S., Gómez-Rubio, V. & Rue, H. Spatial data analysis with R-INLA with some extensions. *J. Stat. Softw.* **63**, 1–31 (2015).

[20] Yin, X., Aiken, J. M., Harris, R. & Bamber, J. L. A Bayesian spatio-temporal model of COVID-19 spread in England. *Sci. Rep.* **14**, 10335 (2024). 38710934 10.1038/s41598-024-60964-0 PMC11074120

[21] Kianfar, N., Mesgari, M. S., Mollalo, A. & Kaveh, M. Spatio-temporal modeling of COVID-19 prevalence and mortality using artificial neural network algorithms. *Spat. Spatiotemp. Epidemiol.* **40**, 100471 (2022). 10.1016/j.sste.2021.100471 PMC8580864 35120681

[22] Musa, S. S. et al. The heterogeneous severity of COVID-19 in African countries: A modeling approach. *Bull. Math. Biol.* **84**, 32 (2022). 35067773 10.1007/s11538-022-00992-x PMC8784278

[23] Ayoub, A. & Cylia, I. Analysis of spatial distribution of COVID-19 mortality in Algeria 2020: A metroplis-adjusted Langevin algorithm fitting approach. *Roa Iktissadia Rev.* **12**, 149–164 (2022).

[24] Lounis, M. Epdemiology of coronavirus disease 2020 (COVID-19) in Algeria. *New Microbes New Infect.* **39**, 100822 (2021). 33251017 10.1016/j.nmni.2020.100822 PMC7683942

[25] Leveau, C. M., Aouissi, H. A. & Kebaili, F. K. Spatial diffusion of COVID-19 in Algeria during the third wave. *GeoJournal* **88**, 1175–1180 (2023). 35261429 10.1007/s10708-022-10608-5 PMC8893245

[26] Dali-Ali, A., Derkaoui, D. K., Zina, M. & Oukebdane, A. Seroprevalence of COVID-19 in Oran: Cross-sectional study. *Microbiol. Spectr.* **11**, e0087623 (2023). 37284756 10.1128/spectrum.00876-23 PMC10433985

[27] Aouissi, H. A. Algeria’s preparedness for omicron variant and for the fourth wave of COVID-19. *Glob. Health Med.* **3**, 413–414 (2021). 35036625 10.35772/ghm.2021.01117 PMC8692091

[28] Khennouchi, N. C. E. H., Meradi, L., Hacini, R., Saighi, R. A. & Yahiaoui, M. Epidemiological state, patient’s characteristics, and COVID-19 vaccination levels in Algeria. *South Fla. J. Health* **4**, 225–240 (2023).

[29] IMF. IMF policy responses to COVID 19. https://www.imf.org/en/Topics/imf-and-covid19/Policy-Responses-to-COVID-19 (2021). Accessed 30 Dec 2024.

[30] Monitoring, G. Global monitoring : COVID-19 pandemic - Algeria. https://global-monitoring.com/gm/page/events/epidemic-0001983.sfkWEJiCejIE.html?lang=en (2021). Accessed 30 Dec 2024.

[31] Kacimi, S. E. O. et al. Determinants of COVID-19 vaccine engagement in Algeria: A population-based study with systematic review of studies from arab countries of the MENA region. *Front. Public Health* **10**, 843449 (2022). 35712268 10.3389/fpubh.2022.843449 PMC9196869

[32] Midoun, M. & Amrani-Midoun, A. Analysis of spatiotemporal pattern for COVID-19 in Algeria using space-time-cubes. *Int. Rev. Model. Simul. (IREMOS)* **15**, 27 (2022).

[33] Naing, N. N. Easy way to learn standardization: direct and indirect methods. *Malays. J. Med. Sci.* **7**, 10–15 (2000). 22844209 PMC3406211

[34] Blangiardo, M., Cameletti, M., Baio, G. & Rue, H. Spatial and spatio-temporal models with R-INLA. *Spat. Spatiotemp. Epidemiol.* **7**, 39–55 (2013). 10.1016/j.sste.2013.07.003 24377114

[35] Martino, S. & Riebler, A. Integrated nested Laplace approximations (INLA). *Wiley StatsRef: Stat. Ref. Online*. 1–19. 10.1002/9781118445112.stat08212 (2020).

[36] Gómez-Rubio, V. *Bayesian Inference with INLA* (Chapman and Hall/CRC, 2020).

[37] Salim, M. F., Satoto, T. B. T. & Danardono. Predicting spatio-temporal dynamics of dengue using INLA (integrated nested Laplace approximation) in Yogyakarta, Indonesia. *BMC Public Health* **25**, 1321 (2025). 10.1186/s12889-025-22545-2 PMC11977928 40200204

[38] Satorra, P. & Tebé, C. Bayesian spatio-temporal analysis of the COVID-19 pandemic in Catalonia. *Sci. Rep.* **14**, 4220 (2024). 38378913 10.1038/s41598-024-53527-w PMC10879174

[39] Simpson, D., Rue, H., Riebler, A., Martins, T. G. & Sørbye, S. H. Penalising model component complexity: A principled, practical approach to constructing priors. *Stat. Sci.* **32**, 1–28 (2017).

[40] Knorr-Held, L. Bayesian modelling of inseparable space-time variation in disease risk. *Stat. Med.* **19**, 2555–2567 (2000). 10960871 10.1002/1097-0258(20000915/30)19:17/18<2555::aid-sim587>3.0.co;2-#

[41] Ugarte, M. D., Adin, A., Goicoa, T. & Militino, A. F. On fitting spatio-temporal disease mapping models using approximate Bayesian inference. *Stat. Methods Med. Res.* **23**, 507–530 (2014). 24713158 10.1177/0962280214527528

[42] R-INLA. R-INLA Project. https://www.r-inla.org/home (2015). Accessed 30 Dec 2024.

